# Effectiveness of Electronic Quality Improvement Activities to Reduce Cardiovascular Disease Risk in People With Chronic Kidney Disease in General Practice: Cluster Randomized Trial With Active Control

**DOI:** 10.2196/54147

**Published:** 2025-02-03

**Authors:** Jo-Anne Manski-Nankervis, Barbara Hunter, Natalie Lumsden, Adrian Laughlin, Rita McMorrow, Douglas Boyle, Patty Chondros, Shilpanjali Jesudason, Jan Radford, Megan Prictor, Jon Emery, Paul Amores, An Tran-Duy, Craig Nelson

**Affiliations:** 1 Primary Care and Family Medicine Lee Kong Chian School of Medicine Singapore Singapore; 2 Centre for Research Excellence in Interactive Digital Technology to Transform Australia’s Chronic Disease Outcomes Prahan Australia; 3 Department of General Practice and Primary Care University of Melbourne Melbourne Australia; 4 Western Health Chronic Disease Alliance Western Health Sunshine Australia; 5 Department of General Practice University College Cork Cork Ireland; 6 Central Northern Adelaide Renal and Transplantation Service Royal Adelaide Hospital University of Adelaide Adelaide Australia; 7 Launceston Clinical School University of Tasmania Launceston Australia; 8 Melbourne Law School University of Melbourne Melbourne Australia; 9 Centre for Health Policy, Melbourne School of Population and Global Health University of Melbourne Melbourne Australia; 10 Australian Centre for Accelerating Diabetes University of Melbourne Melbourne Australia; 11 Department of Medicine University of Melbourne Sunshine Australia; 12 Department of Nephrology Western Health Sunshine Australia

**Keywords:** primary care, general practice, clinical decision support, chronic kidney disease, cardiovascular disease, cardiovascular, clinical decision, decision support, support, kidney, kidney disease, electronic medical record, risk, risk reduction, pharmacological, pharmacological therapy, medical records, logistic model

## Abstract

**Background:**

Future Health Today (FHT) is a program integrated with electronic medical record (EMR) systems in general practice and comprises (1) a practice dashboard to identify people at risk of, or with, chronic disease who may benefit from intervention; (2) active clinical decision support (CDS) at the point of care; and (3) quality improvement activities. One module within FHT aims to facilitate cardiovascular disease (CVD) risk reduction in people with chronic kidney disease (CKD) through the recommendation of angiotensin-converting enzyme inhibitor inhibitors (ACEI), angiotensin receptor blockers (ARB), or statins according to Australian guidelines (defined as appropriate pharmacological therapy).

**Objective:**

This study aimed to determine if the FHT program increases the proportion of general practice patients with CKD receiving appropriate pharmacological therapy (statins alone, ACEI or ARB alone, or both) to reduce CVD risk at 12 months postrandomization compared with active control (primary outcome).

**Methods:**

General practices recruited through practice-based research networks in Victoria and Tasmania were randomly allocated 1:1 to the FHT CKD module or active control. The intervention was delivered to practices between October 4, 2021, and September 30, 2022. Data extracted from EMRs for eligible patients identified at baseline were used to evaluate the trial outcomes at the completion of the intervention period. The primary analysis used an intention-to-treat approach. The intervention effect for the primary outcome was estimated with a marginal logistic model using generalized estimating equations with robust SE.

**Results:**

Overall, of the 734 eligible patients from 19 intervention practices and 715 from 21 control practices, 82 (11.2%) and 70 (9.8%), respectively, had received appropriate pharmacological therapy (statins alone, ACEI or ARB alone, or both) at 12 months postintervention to reduce CVD risk, with an estimated between-trial group difference (Diff) of 2.0% (95% CI –1.6% to 5.7%) and odds ratio of 1.24 (95% CI 0.85 to 1.81; *P*=.26). Of the 470 intervention patients and 425 control patients that received a recommendation for statins, 61 (13%) and 38 (9%) were prescribed statins at follow-up (Diff 4.3%, 95% CI 0 to 8.6%; odds ratio 1.55, 95% CI 1.02 to 2.35; *P*=.04). There was no statistical evidence to support between-group differences in other secondary outcomes and general practice health care use.

**Conclusions:**

FHT harnesses the data stored within EMRs to translate guidelines into practice through quality improvement activities and active clinical decision support. In this instance, it did not result in a difference in prescribing or clinical outcomes except for small changes in statin prescribing. This may relate to COVID-19–related disruptions, technical implementation challenges, and recruiting higher performing practices to the trial. A separate process evaluation will further explore factors impacting implementation and engagement with FHT.

**Trial Registration:**

ACTRN12620000993998; https://www.anzctr.org.au/Trial/Registration/TrialReview.aspx?id=380119

## Introduction

More than 4 in 5 Australians visit their general practice at least once per year and 2 million attend every week [1,2]. As medical knowledge continues to increase exponentially, it is crucial that this knowledge is translated into practice efficiently and effectively. Australian general practitioners (GPs) need to have a good working knowledge of 167 conditions to manage 85% of presentations [3], but there is no way for GPs to easily keep up to date with a multitude of guidelines that are stored in different locations while delivering person-centered care. This is critically important for people at risk of, or with, chronic diseases such as chronic kidney disease (CKD), where early detection and management have the potential to reduce disease progression and the development of complications, improve quality of life, and reduce the burden on the health care system [4].

CKD is a common condition, primarily managed in the general practice setting, that is defined as kidney damage reduced function, or both present for at least 3 months [5]. In Australia, CKD affects up to 2 million people and costs the Australian economy more than Aus $5 billion per year (a currency exchange rate as of October 1, 2021, of Aus $1=US $0.726 is applicable) [6]. Untreated CKD can lead to end-stage kidney failure, which may require dialysis or transplant therapies, but the most common cause of death in people with CKD is cardiovascular disease (CVD) [4]. Kidney Health Australia guidelines support the detection of CKD in high-risk populations and recommend pharmacological treatment with angiotensin-converting enzyme inhibitor inhibitors (ACEI) or angiotensin receptor blockers (ARB), or statins or both statins and an ACEI or ARB to reduce CKD progression and CVD risk [5]. It has been identified that additional strategies are required to optimize CVD risk management in general practice [7,8].

Quality improvement (QI) and clinical decision support (CDS) software may help to optimize the care provided to people with CKD in general practice. Building on systematic review evidence for effective elements of these interventions [9,10], we collaborated with GPs, general practice nurses, practice managers, and patients to codesign Future Health Today (FHT) [11]. The FHT software program integrates with the electronic medical records (EMRs) used by over 90% of Australian general practices. It consists of 4 components: a dashboard, a CDS tool, access to resources and QI activities.

This trial aimed to determine whether the FHT platform, packaged with QI support and a series of webinars provided using the Extension for Community Healthcare Outcomes (Project ECHO) program [12] through the Zoom videoconferencing platform, was effective in increasing the proportion of patients with CKD receiving guideline-concordant care to reduce CVD risk at 12 months postrandomization in intervention practices compared with control practices which received an FHT module focused on cancer care. This is a timely trial as policy informing the use of CDS in Australian general practice is currently under development [13].

## Methods

### Overview

We conducted a stratified cluster randomized head-to-head trial with general practices randomly assigned 1:1 to the FHT CKD program or an active control (FHT cancer program). The full protocol was published [14] and registered with the Australia and New Zealand Clinical Trial Registry (ACTRN12620000993998) [15].

### Setting

The FHT trial was conducted in 39 general practices in Victoria and 1 practice in Tasmania, Australia. In Australia, general practice provides continuous, longitudinal care across the lifespan. The Australian government funds general practice through the Medicare Benefit Schedule (MBS) as a fee-per-service model. The MBS is a list of the medical services for which the Australian Government will make a payment [16], although this may not cover the full cost of the consultation.

The COVID-19 pandemic had a significant impact on general practices, service provision, and patient access during the study period. The government strategy was COVID-19 elimination with frequent lockdowns and border closures. The sixth lockdown in Victoria lasted from August 5, 2021, to October 22, 2021, crossing over the commencement of this trial, which had already been delayed by 6 months due to the pandemic. Significant workload related to COVID-19 infection control procedures and vaccination [17], a shift to telehealth [18], and a significant reduction in pathology services utilization were observed [19].

### Recruitment

Forty general practices were recruited between October 2020 and August 2021. Practices were recruited through the VicREN practice-based research and education network at the Department of General Practice and Primary Care, The University of Melbourne [20], the University of Tasmania’s Northern Tasmania General Practice-based research network, and through newsletters sent to practices through Primary Health Networks. Following the referral of interested practices by these networks to the study team, the research team liaised with general practice staff to explain the study further and to arrange consent and the completion of an information technology checklist to ensure that the general practice technology infrastructure that was consistent with the requirements of the FHT software.

General practice eligibility criteria are summarized in Textbox 1. Practices needed to have at least 2500 active patients in their EMR system (ie, not marked as deceased or no longer attending the practice) and use either the Best Practice or Medical Director EMR systems. These 2 systems are estimated to be used by more than 90% of Victorian general practices [21]. Practices also needed to agree to install the GRHANITE data extraction tool [22] so that they could contribute deidentified data from their EMR to the Patron dataset [23] to facilitate analysis of the trial outcome measures.

Practices were compensated Aus $2250 for participating in the trial; additional payments were made to GPs and general practice staff if they were nominated as practice champions (Aus $200) or participated in interviews (Aus $50 per interview).

There was no individual patient-level recruitment or consent required as outcomes were ascertained using deidentified EMR data. Inclusion criteria for patient records: Adults aged 18 to 80 years, inclusive, that were not marked as inactive or deceased, with a recorded diagnosis or pathology tests consistent with CKD as defined by Kidney Health Australia [5] that may benefit from pharmacological therapy to reduce CVD risk at baseline. Individuals with a recorded history of renal transplant or chronic dialysis were excluded. Pregnant individuals were also excluded.

**General practice eligibility criteria.**

**Inclusion criteria**

- Agreed to install the GRHANITE data extraction tool [22] to contribute deidentified data from their electronic medical records (EMRs) to the Patron dataset [23].
- Consultations with at least 35 adults aged ≥18 years per day, >2500 active adult patients (defined as patients who attended the general practice at least 3 times in the last 2 years and not marked as deceased) recorded in their EMR or have at least 50 patients that fit cohort definitions for people with chronic kidney disease not on optimal medications and abnormal test results and additional clinical features placing them at risk of an undiagnosed cancer.
- Employed a general practice nurse.
- Contributed (or willing to contribute) data to the Patron dataset, a repository of data from EMR shared by general practices and curated by the University of Melbourne.
- Used Best Practice or Medical Director EMR software to record clinical consultations, prescription of medications, and ordering and receiving pathology results.
- Could identify a workstation (i5/i7 and 16GB RAM or upgradeable to 16GB) with Windows 10 (operating system) that would have GRHANITE, the data extraction tool, installed.
- Computers had Edge or Chrome web browsers installed.

**Exclusion criteria**

- Had previously participated in other Future Health Today projects.
- Intended to change to another EMR software during the trial period.
- Used a cloud-based EMR system.

### Randomization

General practices were allocated 1:1 to the FHT CKD program or the FHT cancer program (active control), using a computer-generated schedule, stratified by Index of Relative Socioeconomic Disadvantage (IRSD) terciles [24] and the number of full-time equivalent (FTE) GPs (4 or fewer vs greater than 4), using random permuted block sizes of 2 and 4 within the stratum. Practices were randomly allocated after they were all recruited, and practices’ baseline measures were collected.

The random allocation schedule was generated by the statistician not involved in the practice recruitment or data collection. After practices were recruited, the statistician randomly allocated the deidentified practices to one of the 2 intervention groups and then informed the clinical liaison staff, who advised practices of their allocated group. Practices were informed that it was a pragmatic cluster randomized controlled trial with 2 active groups.

Blinding of general practice participants and the team providing QI support was not possible. The statistician conducting the analysis and study investigators not involved in practice support and engagement remained blinded, and allocation of practices was only revealed after the data were analyzed and results interpreted.

### Intervention

#### Future Health Today Software

**Figure 1:**
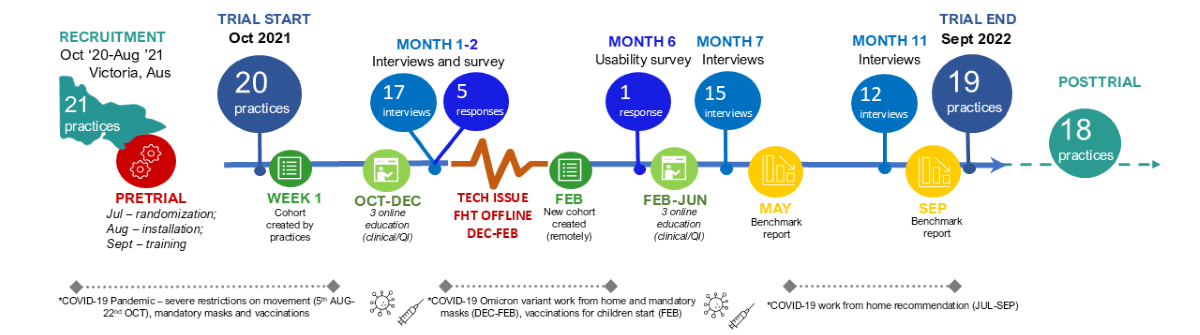
Trial timeline and data sources (chronic kidney disease group).

FHT is software installed in general practices to identify people who may benefit from the implementation of guideline-informed care. There are two main components: (1) a dashboard that can be used to create cohorts of patients that may benefit from review and potential optimization of care and (2) a CDS tool that deploys when a patient file is opened. Login details and passwords were provided for the dashboard. The CDS tool automatically deployed when a patient file was opened within the EMR without the need for the clinician to log in to FHT. FHT cohorts and CDS prompts are generated each night when code related to best practice guideline recommendations is run over EMR systems in the practice. Screenshots that illustrate the different components of FHT are provided in Multimedia Appendix 1. Implementation activities and timing for delivery are detailed in Figure 1. Following quality assurance testing of the FHT platform and the module algorithms in a virtual environment, FHT was installed in all practices (intervention and control) before the commencement of the trial. Practices were asked to install FHT on at least one GP and one practice nurse computer. A training FHT type 2 diabetes module was deployed and available to both intervention and control practices for approximately 4 weeks before the trial, to ensure that FHT was working correctly from a technical perspective. Pretrial training sessions covering the functionality of the FHT CDS and dashboard components were offered to all practices.

At trial commencement, the training FHT type 2 diabetes module was deactivated and the FHT CKD module was deployed to intervention practices. The CKD module consisted of 3 recommendations for medications that aim to reduce cardiovascular disease risk: “Consider initiation of ACEI or ARB,” “Consider initiation of statin for CKD management,” and “Consider initiation of ACEI or ARB (note: BP in target)” for patients who had an indication for these medications but had not been prescribed them. These recommendations were based on guidelines from Kidney Health Australia [5], the Royal Australian College of General Practitioners [25], and the National Vascular Disease Prevention Alliance [26] and were applied to patients who had a recorded diagnosis of CKD or pathology consistent with CKD, as defined by Kidney Health Australia guidelines [5]. Practices were asked to create a cohort of patients with these recommendations in the dashboard at baseline and asked to use FHT as they chose for the following 12 months.

#### Quality Improvement Supports

A practice liaison staff member was assigned to support the intervention practices. This staff member provided software training to practices, and 2 benchmarking reports (6 months and 12 months from trial start) and offered practice guidance in undertaking a quality improvement clinical audit activity related to the intervention. The benchmarking reports summarized the number of patients with recommendations for a specific practice and then compared these to the overall intervention cohort. The practice liaison also coordinated a series of webinars using the Project ECHO program [12], an evidence-based platform that facilitates case-based learning networks with primary care, facilitated by academic medicine departments, and delivered by the Zoom videoconferencing platform. Three separate 1-hour sessions were delivered with a focus on CKD management and reduction of cardiovascular disease risk, and a further three 1-hour sessions were delivered on how to conduct quality improvement activities. The clinical audit and webinar series were accredited by the Royal Australian College of General Practitioners and the Australian College of Rural and Remote Medicine as continuing professional development (CPD) activities. All participants, including nonclinical staff, were provided with certificates of participation in these activities.

Two deviations from the initially proposed protocol occurred, both of which related to the availability of the FHT software, (1) there was a pause in the provision of the CKD module in FHT software from December 16, 2021, to February 14, 2022, due to complexity of the FHT module algorithms and mappings from CKD not being appropriately implemented within the FHT code. This meant that the software was not available for the full intended 12 months, (2) one practice did not have access to FHT modules until 2 weeks after the trial started due to challenges with software installation.

### Consumer Involvement

A consumer advisory group and general practice advisory group (consisting of GPs, general practice nurses, and practice managers) provided input to the trial intervention and interpretation of the trial results.

### Endpoints and Data Collection

All outcomes were measured at 12 months postrandomization. The primary outcome was the proportion of eligible patients with a prescription of ACE inhibitors, ARBs statins, or both according to guidelines. Patients were considered to have received guideline-concordant care if they had a recorded prescription for the classes of medication indicated by FHT recommendations within the study period. If a patient received recommendations for both classes of medication at baseline, for the primary outcome, they were required to have been prescribed both classes of medication. If a prescription was not recorded in the EMR, patients were considered not to have received guideline-concordant care. The secondary outcomes related to the prescription of individual classes of these medications, pathology results (including estimated glomerular filtration rate, albumin creatinine ratio, and cholesterol), and systolic blood pressure; refer to Multimedia Appendix 2 for additional details. The number and billing of general practice consultations were also captured. Costs to the government associated with consultations occurring during the trial were based on the Medicare Benefits Schedule (MBS) rebate for the service items retrieved from the Patron database [27].

Baseline and outcome measures were obtained using patient data extracted from general practice EMRs and stored in the Patron database [28]. Patron data are stored at the University of Melbourne and were only accessible by the study statisticians.

Practice characteristics, including the number of GPs and other general practice staff, billing method, and regionality were collected through a survey before randomization.

We also collected survey and qualitative data relating to implementation, to be reported as a process evaluation elsewhere.

### Sample Size

We determined that 2580 patients from 20 practices per group are required to detect an absolute 10% increase in the difference in the percentage of patients on optimal pharmacological management in the intervention group at 12 months postintervention compared with the control group at 80% power for a 2-sided 5% significance level. Based on electronic health records from 77 general practices that contribute data to the Patron dataset in Victoria, Australia [28], we expected 55% of active control patients with CKD would be on optimal pharmacological management with an intracluster correlation (ICC) coefficient of 0.03 to account for clustering at the practice level. Further, we expected on average 139 eligible patients per practice (fixed cluster size), with a coefficient of variation of 0.41 to account for variable cluster sizes across the practices. The target of 40 practices included 4 additional practices to allow for attrition by 12 months (eg, practice closure, withdrawals, mergers) and the addition of one extra practice per group, for a degree of freedom correction. The effect size of 10% was considered a minimally important difference and worth implementing and is consistent with another Australian general practice study that aimed to improve CVD risk management, albeit not limited to patients with CKD [29].

### Statistical Analysis

Descriptive statistics were used to summarize general practice, clinician, and patient characteristics by study group. The primary analysis used an intention-to-treat approach with practices analyzed according to their allocated group. All general practice patients were included in the analysis, regardless of the occurrence of intercurrent events (eg, death, pregnancy). For the primary outcome, the OR and between-group differences in proportions were estimated with a generalized linear regression model, with the logit and identity link functions, respectively. Generalized estimating equations (GEEs) were used with an exchangeable correlation structure and robust SE to allow for the correlation of outcomes within general practice. The ICC was estimated using a one-way analysis of variance. Binary secondary outcomes used the same method as the primary outcome. For continuous outcomes, a linear mixed effects model was used to estimate the between-group difference in mean change scores with random effects for general practice and individual and adjusted for their respective baseline measure with study group means at baseline constrained to be equal. A proportional odds logistic regression model with GEEs and robust SE was used to estimate cumulative OR for the albumin creatinine ratio level at 12 months. The between-group difference in the rate of consultations per year and rate ratio of visits were modeled using a negative binomial mixed effects model, with random effects for general practice, with GEEs and robust SE.

A 2-part model consisting of a logistic regression model for the probability of positive Medicare benefit and a generalized linear model with a log link and gamma distribution for the positive Medicare benefit was used to assess the association between the intervention and the Medicare benefit. The marginal effect of the intervention was computed in both the logistic regression model (as percentage point change) and the generalized linear regression model (as dollar change).

All regression models included the study group and randomization stratification factors (GP, FTE, and IRSD tercile) as covariates. For secondary binary outcomes, the recommendation to initiate statin and ACEI or ARB was adjusted for the recommendation to consider ACEI or ARB and statin initiation, respectively. Sensitivity analyses included (1) complete cases only (for continuous outcomes, if the linear mixed effects model failed to converge, linear regression with robust SE was used) and (2) adjusting for patient age and sex and previous practice involvement in formalized QI programs. For the binary outcomes, if the model used to estimate the risk difference failed to converge, the additional covariates were excluded (noted in the results tables). Adherence-adjusted analysis was not conducted as we were unable to measure adherence to the intervention.

Estimated intervention effects were reported with 95% CI and *P* values. No corrections were made for multiple comparisons.

All analyses were conducted using Stata 17.0 (StataCorp). The full statistical analysis plan has been uploaded to the ANZCTR.

### Ethical Considerations

The FHT trial was approved by the Faculty of Medicine, Dentistry and Health Sciences Human Ethics Sub-Committee at the University of Melbourne (Ethics ID:2056564). General practices signed a study agreement to participate in the trial. Patients were not recruited for this study, with data from the Patron dataset used to calculate the primary and secondary trial outcomes. The Patron dataset has a waiver of consent granted by the Faculty of Medicine, Dentistry and Health Sciences Human Ethics Sub-Committee at the University of Melbourne (ethics number 2023-23358-37538-6). Data within the Patron dataset is anonymized. General practices were paid Aus $2250 to compensate them for any IT support, meeting or training attendance, generating patient lists, mailouts, monthly key staff informant interviews, and participation in quality improvement activities and ECHO education activities.

## Results

### Participating Practices

**Figure 2:**
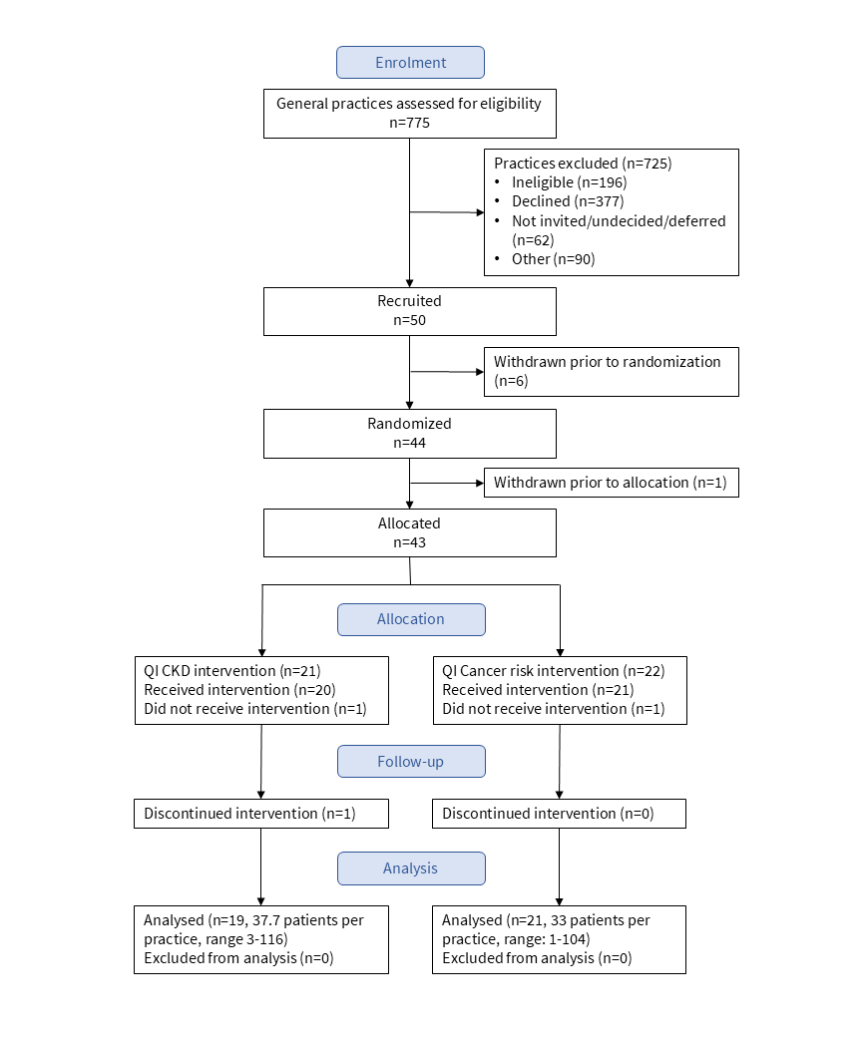
Practice recruitment, allocation, follow-up, and data analysis.

Between October 2020 and August 2021, there were 775 general practices assessed for eligibility, following which 50 practices were recruited to participate in the trial (refer to Figure 2). Six practices withdrew before randomization (all these practices were owned by the same group), and a practice withdrew after randomization but before they were notified of their group allocation. Two randomized practices did not receive the allocated intervention and 1 discontinued the intervention during the trial, leaving forty participating practices that completed the trial. Patron data was only provided for the forty practices that completed the study.

Baseline characteristics of the 40 practices, clinicians, and staff (Table 1) and their eligible patients (Table 2) were balanced between the study groups. A total of 734 and 715 eligible patients in the intervention and control groups, respectively, were identified in the EMR with a recorded diagnosis or pathology tests consistent with CKD that may benefit from pharmacological therapy to reduce CVD risk. Of these, 17% had a recommendation for both ACEI or ARB and satins, 45% for statins only, and the remaining 38% for ACEI/ARBS only.

**Table 1:** Baseline characteristics of practices, clinicians, managers, and staff, overall and by study group.

				All practices (N=40)		Intervention group (N=19)		Control group (N=21)
**Practice characteristics**								
	**State, n (%)**							
		Victoria	39 (97.5)		19 (100)		20 (95.2)	
		Tasmania	1 (2.5)		0 (0)		1 (4.8)	
	**Relative socioeconomic disadvantage index (terciles), n (%)**							
		1 (most disadvantaged)	12 (30)		6 (31.6)		6 (28.6)	
		2	12 (30)		6 (31.6)		6 (28.6)	
		3 (least disadvantaged)	16 (40)		7 (36.8)		9 (42.8)	
**Practice size**								
	4 or fewer FTE^a^ GPs^b^, n (%)			21 (52.5)		9 (47.4)		12 (57.1)
	Greater than 4 FTE GPs, n (%)			19 (47.5)		10 (52.6)		9 (42.9)
	Previous quality improvement program participation, n (%)			18 (45)		9 (47.4)		9 (42.9)
	Eligible patients per practice, mean (range)			35.3 (1-116)		37.7 (3-116)		33.0 (1-104)
	Workstations with Future Health Today per practice, mean (range)^c^			6.4 (1-20)		7.4 (1-20)		5.5 (1-19)
	Future Health Today users per practice, mean (range)			31.8 (10-160)		30.2 (14-73)		33.2 (10-160)
	Number of FTE GPs, median (IQR)			4.5 (3.5-6.0)		4.5 (3.5-7.5)		4.0 (3.5-5.5)
	Number of FTE practice nurses, median (IQR)			1.8 (1.0-2.5)		1.5 (1.0-3.0)		2.0 (1.5-2.5)
	Number of FTE practice managers or administrative staff, median (IQR)			4.0 (3.0-5.0)		4.5 (3.5-6.0)		3.5 (2.5-4.5)
**General practitioners**				309		164		145
	**Sex, n (%)**							
		Male	178 (57.6)		93 (56.7)		85 (58.6)	
		Female	131 (43.4)		71 (43.3)		60 (41.4)	
	**Age, n (%)**							
		<35 years	40 (15)		19 (14.2)		21 (15.8)	
		35-50 years	129 (48.3)		56 (41.8)		73 (54.9)	
		>50 years	98 (36.7)		59 (44)		39 (29.3)	
**Nurses**				123		64		59
	**Sex, n (%)**							
		Male	7 (5.7)		3 (4.7)		4 (6.8)	
		Female	116 (94.3)		61 (95.3)		55 (93.2)	
	**Age^d^, n (%)**							
		<35 years	60 (49.2)		33 (52.4)		32 (50)	
		35-50 years	31 (25.4)		14 (22.2)		17 (26.6)	
		>50 years	31 (25.4)		16 (25.4)		15 (23.4)	
**Practice managers or administrative staff**				257		130		127
	**Sex, n (%)**							
		Male	27 (10.5)		14 (10.8)		13 (10.2)	
		Female	230 (89.5)		116 (89.2)		114 (89.8)	
	**Age, n (%)**							
		<35 years	130 (51.6)		75 (60)		55 (43.3)	
		35-50 years	59 (23.4)		26 (20.8)		33 (26)	
		>50 years	63 (25)		24 (19.2)		39 (30.7)	

^a^FTE: full-time equivalent.

^b^GPs: general practitioner.

^c^Six intervention and 2 control group practices ran a “terminal server” that pushed Future Health Today to all workstations.

^d^Percentages for age categories were calculated based on the number of staff who provided age data.

**Table 2:** Baseline characteristics of patients, overall and by study group.

Patient characteristics		All participants (N=1449)	Intervention group (n=734)	Control group (n=715)
**Sex, n (%)**				
	Male	812 (56)	402 (54.8)	410 (57.3)
	Female	637 (44)	332 (45.2)	305 (42.7)
Age (years), mean (SD)		65.9 (12.1)	66.3 (11.3)	65.4 (12.9)
**Prescribed ACEI^a^** **or ARB^b^** **, n (%)**				
	ACEI only	215 (14.8)	109 (14.9)	106 (14.8)
	ARB only	321 (22.2)	166 (22.6)	155 (21.7)
	ACEI and ARB	6 (0.4)	4 (0.6)	2 (0.3)
	None	907 (62.6)	455 (62)	452 (63.2)
**Prescribed statins, n (%) **				
	Yes	424 (29.3)	192 (26.2)	232 (32.5)
	No	1025 (70.7)	542 (73.8)	483 (67.5)
**Guideline recommended initiation, n (%)**				
	Both ACEI or ARB and statins	246 (17)	131 (17.9)	115 (16.1)
	ACEI or ARBs only	554 (38.2)	264 (36)	290 (40.5)
	Statin only	649 (44.8)	339 (46.2)	310 (43.4)
**Cardiovascular disease risk category, n (%)**				
	Total sample	1065 (73.5)	539 (73.4)	526 (73.8)
	Low	85 (8)	43 (7.6)	42 (8)
	Moderate	29 (2.7)	14 (2.7)	15 (2.9)
	High	951 (89.3)	482 (89.7)	469 (89.2)
Type 2 diabetes n (%)		633 (43.7)	308 (42)	325 (45.5)
**Systolic blood pressure** **(mmHg)**				
	Number (%)	843 (58.2)	434 (59.1)	409 (57.2)
	Mean (SD)	138 (20)	138 (20)	137 (19)
**Lipids (all mmol/L) **				
	Total cholesterol, n (%)	916 (63.2)	465 (63.4)	451 (63.1)
	Total cholesterol, mean (SD)	4.8 (1.2)	4.8 (1.2)	4.8 (1.3)
	Low-density lipoproteins, n (%)	844 (58.2)	431 (58.7)	413 (57.8)
	Low-density lipoproteins, mean (SD)	2.7 (1.1)	2.7 (1.1)	2.6 (1.1)
	High-density lipoproteins, n (%)	865 (59.7)	444 (60.5)	421 (58.9)
	High-density lipoproteins, mean (SD)	1.3 (0.4)	1.3 (0.4)	1.3 (0.4)
	Triglycerides, n (%)	914 (63.1)	464 (63.2)	450 (62.9)
	Triglycerides mean (SD)	2.1 (1.8)	2.0 (1.4)	2.1 (2.1)
**Urine albumin-creatinine ratio (mg/mmol)^c^**				
	Number (%)	702 (48.4)	323 (44)	379 (53)
	Median (IQR)^c^	5.5 (1.6-21.7)	5.2 (1.4-22.5)	5.6 (1.7-20.2)
	Macroalbuminuria, n (%)	137 (19.5)	64 (19.8)	73 (19.3)
	Microalbuminuria, n (%)	343 (48.9)	150 (46.4)	193 (50.9)
	Normal, n (%)	222 (31.6)	109 (33.8)	113 (29.8)
**Estimated glomerular filtration rate (mL/min/1.73m^2^)**				
	Number (%)	1175 (81.1)	592 (80.7)	583 (81.5)
	Mean (SD)	55.3 (20.9)	53.5 (20.4)	57.1 (21.2)
	G1 (≥90), n (%)	149 (12.7)	61 (10.3)	98 (15.1)
	G2 (60-89), n (%)	224 (19.1)	105 (17.7)	119 (20.4)
	G3a (45-59), n (%)	428 (36.4)	221 (37.3)	207 (35.5)
	G3b (30-44), n (%)	267 (22.7)	146 (24.7)	121 (20.8)
	G4 (15-29), n (%)	73 (6.2)	37 (6.3)	36 (6.2)
	G5 (<15), n (%)	35 (2.9)	22 (3.7)	12 (2.1)

^a^ACEI: angiotensin-converting enzyme inhibitor.

^b^ARB: angiotensin receptor blocker.

^c^Median and IQR were reported due to skewed distribution.

### Primary and Secondary Outcomes

#### Primary Outcome

Of patients identified as eligible for pharmacological therapy to reduce CVD risk, 11.2% (82/734) in the intervention group and 9.8% (70/715) in the control group had received appropriate pharmacological therapy at 12 months post-intervention, an increase of 2.0% (95% CI –1.6% to 5.7%) (Table 3), with the confidence bounds excluding our hypothesized minimally important difference of 10%. The ICC for the proportion of patients who were prescribed ACEI or ARB and Statin at baseline was estimated as 0.03 (95% CI 0.01 to 0.05).

**Table 3:** Primary outcome and secondary outcomes related to medication prescriptions

				All participants, n (%)		Intervention group, n (%)		Control group, n (%)		Estimated effect size (95% CI)					*P* value
										Diff^a^ (95% CI), %		OR^b^ (95% CI)			
**Primary outcome**															
	**Angiotensin-converting enzyme inhibitor or angiotensin receptor blocker and statins**														
		Number of participants	1449		734		715								
		Primary analysis	152 (10.5)		82 (11.2)		70 (9.8)		2.0 (–1.6 to 5.7)		1.24 (0.85 to 1.81)		.26		
		Sensitivity analysis^c^	152 (10.5)		82 (11.2)		70 (9.8)		2.6 (0.0 to 6.1)		1.30 (0.90 to 1.87)		.17		
**Secondary outcomes**															
	**Angiotensin-converting enzyme inhibitor or angiotensin receptor blocker**														
		Number of participants	800		395		405								
		Primary analysis	100 (12.5)		48 (12.2)		52 (12.8)		0.0 (–3.7 to 4.6)		0.94 (0.62 to 1.42)		.76		
		Sensitivity analysis^a^	100 (12.5)		48 (12.2)		52 (12.8)		1.8 (–2.6 to 6.2)		1.04 (0.69 to 1.57)		.85		
	**Statins**														
		Number of participants	895		470		425								
		Primary analysis	99 (11.1)		61 (13)		38 (9)		4.3 (0 to 8.6)		1.55 (1.02 to 2.35)		.04		
		Sensitivity analysis^a^	99 (11.1)		61 (13)		38 (9)		4.4 (0 to 8.5)		1.56 (1.04 to 2.34)		.03		

^a^Diff: difference in mean change score or percentages between the 2 groups.

^b^OR: odds ratio.

^c^Sensitivity analysis 1, as above, with adjustment for participant age and sex and general practitioner participation in formal quality improvement activities.

#### Secondary Outcomes

When we examined the patients with recommendations for statins and ACEI or ARBS, separately, 13% (61/470) of patients in the intervention group received a statin prescription compared to 9% (38/425) in the control group, an estimated between-group difference of 4.3% (95% CI 0%-8.6%). The sensitivity analyses showed similar results. Primary analyses are generalized linear regression using generalized estimating equations with robust SE, adjusted for GP FTE and IRSD tercile.

Table 4 shows that there was no statistical evidence of an intervention effect for mean change in pathology measures from baseline between the intervention and control groups. Primary analyses for continuous measures used a linear mixed effects model with random effects for general practice and individuals, adjusted for baseline measure of the outcome. Primary analyses for binary outcomes used generalized linear regression using generalized estimating equations with robust standard errors. All primary analyses adjusted for GP FTE and IRSD tercile.

Table 5 summarizes the nonpathology measures of secondary outcomes. There was no evidence of an effect of the intervention on systolic blood pressure change, cardiovascular disease risk category, or rate of general practice visits of patients over the 12-month intervention period compared to the control group. Primary analyses for continuous measures used a linear mixed effects model with random effects for general practice and individuals and adjusted for baseline measures of the outcome. Primary analyses for ordinal outcomes used proportional odds logistic regression, using generalized estimating equations with robust standard errors. The primary analysis of the rate of visit data used a negative binomial mixed effects model with random effects for general practice. All primary analyses adjusted for GP FTE and IRSD tercile.

**Table 4:** Secondary outcomes for laboratory pathology measures.

		All participants	Intervention group	Control group	Estimated effect size, (95% CI)			*P* value
**Cholesterol change (mmol/L), mean (SD)**								
	Sample size	563	260	303				
	Primary analysis	0.14 (0.94)	0.07 (0.84)	0.20 (1.01)	Diff^a^ –0.11 (–0.28 to 0.07)			.24
	Sensitivity analysis^b^	0.14 (0.94)	0.07 (0.84)	0.20 (1.01)	Diff –0.09 (–0.27 to 0.09)			.32
	Sensitivity analysis^c,d^	0.14 (0.94)	0.07 (0.84)	0.20 (1.01)	Diff –0.13 (–0.27 to 0.03)			.06
**Low-density lipoproteins change (mmol/L), mean (SD)**								
	Sample size	484	230	254				
	Primary analysis	0.13 (0.75)	0.05 (0.67)	0.20 (0.82)	Diff –0.10 (–0.26 to 0.06)			.21
	Sensitivity analysis^b^	0.13 (0.75)	0.05 (0.67)	0.20 (0.82)	Diff –0.10 (–0.26 to 0.06)			.24
	Sensitivity analysis^c^	0.13 (0.75)	0.05 (0.67)	0.20 (0.82)	Diff –0.13 (–0.26 to 0.00)			.05
**High-density lipoproteins change (mmol/L), mean (SD)**								
	Sample size	518	247	271				
	Primary analysis	0.00 (0.21)	–0.01 (0.20)	0.01 (0.22)	Diff –0.04 (–0.09 to 0.02)			.17
	Sensitivity analysis^b^	0.00 (0.21)	–0.01 (0.20)	0.01 (0.22)	Diff –0.05 (–0.10 to 0.01)			.09
	Sensitivity analysis^c^	0.00 (0.21)	–0.01 (0.20)	0.01 (0.22)	Diff –0.03 (–0.07 to 0.01)			.11
**Triglycerides change (mmol/L), mean (SD)**								
	Sample size	560	258	302				
	Primary analysis	0.07 (1.61)	0.06 (0.88)	0.07 (2.03)	Diff 0.09 (–0.11 to 0.29)			.36
	Sensitivity analysis^b^	0.07 (1.61)	0.06 (0.88)	0.07 (2.03)	Diff 0.15 (–0.05 to 0.35)			.15
	Sensitivity analysis^c^	0.07 (1.61)	0.06 (0.88)	0.07 (2.03)	Diff 0.09 (–0.11 to 0.29)			.39
**Urine albumin-creatinine ratio change (mg/mmol), mean (SD)**								
	Sample size	398	178	220				
	Primary analysis	–2.13 (41.80)	–6.56 (42.99)	1.45 (40.56)	Diff –9.11 (–21.36 to 3.14)			.15
	Sensitivity analysis^b^	–2.13 (41.80)	–6.56 (42.99)	1.45 (40.56)	Diff –8.97 (–21.23 to 3.31)			.15
	Sensitivity analysis^c^	–2.13 (41.80)	–6.56 (42.99)	1.45 (40.56)	Diff –6.69 (–14.33 to 0.95)			.09
**Urine albumin-creatinine ratio 30% reduction**								
	Sample size	398	178	220				
	Primary analysis, n (%)	117 (29.4)	54 (30.3)	63 (28.6)	Diff 3.1% (–5.7% to 12.0%); OR^e^ 1.14 (.74 to 1.76)			.55
	Sensitivity analysis^b^, n (%)	117 (29.4)	54 (30.3)	63 (28.6)	Diff 2.4% (–6.8% to 11.5%); OR 1.09 (.69 to 1.73)			.70
**Estimated glomerular filtration rate change (mL/min/1.73^2^), mean (SD)**								
	Sample size	894	437	457				
	Primary analysis	0.10 (8.47)	–0.27 (8.30)	0.44 (8.64)	Diff 1.35 (–1.15 to 3.86)			.29
	Sensitivity analysis^b^	0.10 (8.47)	–0.27 (8.30)	0.44 (8.64)	Diff 1.04 (–1.37 to 3.45)			.40
	Sensitivity analysis^c^	0.10 (8.47)	–0.27 (8.30)	0.44 (8.64)	Diff –0.56 (–1.75 to 0.64)			.36

^a^Diff: difference in mean change score or percentages between the 2 groups.

^b^Sensitivity analysis 1 additionally adjusts for participant age and sex, and practice participation in formal QI activities.

^c^Sensitivity analysis 2 using complete case only for outcomes with missing data.

^d^Linear regression model with robust SE used as an alternative to linear mixed effects model due to convergence issues.

^e^OR: odds ratio.

**Table 5:** Secondary outcomes for nonpathology measures.

			All participants		Intervention group		Control group		Estimated effect size (95% CI)			*P* value
**Systolic blood pressure** **changes from baseline mmHg, mean (SD)**												
	Sample size	676		335		341						
	Primary analysis	2.29 (20.26)		3.11 (20.66)		1.47 (19.86)		Diff^a^ 0.75 (–1.71 to 3.21)			.55	
	Sensitivity analysis^b^	2.29 (20.26)		3.11 (20.66)		1.47 (19.86)		Diff 0.88 (–1.58 to 3.35)			.49	
	Sensitivity analysis^c^	2.29 (20.26)		3.11 (20.66)		1.47 (19.86)		Diff 1.00 (–1.49 to 3.48)			.43	
**Cardiovascular d** **isease** **risk category,** **n (%)**												
	Sample size	1019		516		503						
	Primary analysis	—^d^		—		—		Cumulative OR^e^ 0.89 (0.64 to 1.22)			.47	
	Low CVD^f^ risk	101 (9.9)		48 (9.3)		53 (10.5)						
	Medium CVD risk	39 (3.8)		23 (4.5)		16 (3.2)						
	High CVD risk	879 (86.3)		445 (86.2)		434 (86.3)						
	Sensitivity analysis^b^	—		—		—		Cumulative OR 0.81 (0.60 to 1.10)			.18	
**Rate of visits, n (%)**												
	Sample size	1447		734		713						
	Number of visits, mean (SD)	17,237		7786		9451						
	Primary analysis	11.91 (14.45)		10.61 (12.16)		13.26 (16.37)		Diff –0.14 (–0.38 to 0.11); RR^g^ 0.87 (0.68 to 1.11)			.27	
	Sensitivity analysis^b^	11.91 (14.45)		10.61 (12.16)		13.26 (16.37)		Diff –0.15 (–0.38 to 0.08); RR 0.86 (0.69 to 1.08)			.20	

^a^Diff: difference in mean change score or percentages between the 2 groups.

^b^Sensitivity analysis 1 additionally adjusts for participant age and sex and practice participation in formal quality improvement activities.

^c^Sensitivity analysis 2 using complete cases for outcomes with missing data.

^d^Not applicable.

^e^OR: odds ratio.

^f^CVD: cardiovascular disease.

^g^RR: rate ratio.

### Health Care Costs

Table 6 summarizes the health economic outcomes. Of 734 patients in the intervention group, 541 (73.7%) had at least one general practice consultation associated with an MBS rebate, compared to 80.6% (576/715) patients in the control group. Patients meeting the criteria for the CKD FHT module in the practices receiving the intervention had a 6.7 percentage point lower probability of having at least one general practice consultation associated with an MBS rebate compared to those in the control group, but there was no evidence of a difference (95% CI –0.26 to 0.13).

Patients in the intervention group who claimed at least one MBS rebate during the trial period had Aus $38.55 lower Medicare benefits compared with the control group over 12 months, roughly equivalent to one standard consultation in general practice lasting up to 20 minutes. There was no evidence of a difference between groups (95% CI Aus $328.46 to Aus $251.35; *P*=0.79). Primary analyses for binary outcomes used logistic regression, with robust SE. Primary analysis of positive Medicare benefits was carried out with a generalized linear model with log link and gamma distribution, with robust standard errors. All primary analyses adjusted for GP FTE and IRSD tercile.

**Table 6:** Health economic outcomes.

			All participants		Intervention group		Control group		Estimated effect size (95% CI)			*P* value
**Probability of incurring Medicare Benefits Schedule rebate (logistic regression), n (%)**												
	Sample size	1449		734		715						
	Primary analysis	1117 (77.1)		541 (73.7)		576 (80.6)		Diff^a^ –0.067 (–0.260 to 0.125); odds ratio 0.719 (0.285 to 1.82)			.49	
	Sensitivity analysis^b^	1117 (77.1)		541 (73.7)		576 (80.6)		Diff –0.094 (–0.337 to 0.148; odds ratio 0.659 (0.236 to 1.84)			.45	
**Positive Medicare Benefits Schedule rebate (generalized linear model), Aus $, mean (SE)**												
	Sample size	1117		541		576						
	Primary analysis	847.28 (23.01)		850.96 (33.33)		843.84 (31.81)		Diff –38.555 (–328.463 to 251.353)			.79	
	Sensitivity analysis^b^	847.28 (23.01)		850.96 (33.33)		843.84 (31.81)		Diff 0.880 (–255.905 to 257.666)			.99	

^a^Diff: difference in mean change score or percentages between the 2 groups.

^b^Sensitivity analysis 1 additionally adjusts for participant age and sex and practice participation in formal quality improvement activities (and a dummy variable for missing quality improvement practice participation).

## Discussion

### Principal Results

Our findings do not support that the intervention was effective in increasing the proportion of eligible patients with optimal pharmacological therapies at 12 months postintervention to reduce their CKD risk. Among general practice adult patients identified in the EMR at baseline with a recorded diagnosis or pathology tests consistent with CKD that may benefit from an ACEI or ARB or statin prescription or both to reduce the risk of CKD based on guidelines, we found that 11.2% and 9.8% of eligible patients received appropriate pharmacological therapy at 12 months post-randomization in those receiving the FHT CKD module compared to active control, an estimated 2% difference between the intervention and control groups.

For participants with a recommendation to initiate a statin, 13.0% received a prescription for statin medication in the intervention group compared to 9.0% of control patients, an estimated 4.3% (95% CI 0% to 8.6%) increase in statin prescribing when initiation was recommended. Although the confidence bound excluded the 10% effect size hypothesized as minimally important for optimal pharmacological therapies, the confidence bounds include improvement in statins prescribing when recommended which could potentially impact reducing cardiovascular events [30], particularly when considering the estimated effect size may be attenuated as a result of several extenuating circumstances.

Circumstances that may have impacted the trial findings include: pausing access of practices to the CKD module from December 16, 2021, to February 14, 2022, due to the complexity of the FHT module algorithms and mappings from CKD not being appropriately implemented within the FHT code; challenges with software installation in some practices with one practice not having access to FHT modules until two weeks after trial start; the number of patients identified in the module being approximately half that proposed in the target sample size; and variation in some of the coding implemented in FHT compared with that provided in the business requirements document. A sensitivity analysis conducted on the cohort of patients who would have been identified, had the algorithms been implemented without error, did not change the trial findings (data not shown).

Further, our trial was conducted during the COVID-19 pandemic which included stay-at-home orders and general practices needed to deliver COVID-19 vaccinations. As a result, there were unprecedented workforce pressures that may have impacted the ability of practices to use FHT and reduced patient attendance at general practice. GPs may have had more confidence in prescribing statins than ACEI or ARB as these medications do not require pathology tests postinitiation, which may have been an additional consideration during the pandemic.

### Strengths and Limitations

Our study has several strengths. First, this was a pragmatic trial and so the results that are obtained are likely to reflect the impact of FHT in real-world practice. Forty practices were included in the analysis, although we had anticipated 36 after allowing for attrition. EMR data were used to capture patient data and outcome measures minimizing selection bias. Without this EMR data capture, the trial would have been logistically impossible due to the impact of COVID-19 on the general practice workforce and the COVID-19 restrictions that were in place restricted travel and access to medical practices by researchers during this period. Our light touch intervention was designed to overcome the known financial and scalability barriers to more traditional academic detailing. Finally, randomizing practices rather than individuals minimized the risk of contamination.

Study limitations included cluster sizes being smaller than anticipated and there was a lower percentage of eligible patients receiving appropriate pharmacological therapy at 12 months in both groups than expected. Possible reasons include that we may have attracted higher performing practices to participate in the trial, so there were smaller proportions of patients with CKD who were not being managed according to guidelines; improvements in coding of the EMR data between the datasets used to estimate the parameters to determine the sample size calculations and then used to measure the primary outcome; variation in the quality of recording of data in EMRs within participating general practices; or fewer patients than anticipated may have attended the general practice during the COVID-19 pandemic due to the Victorian lockdowns. However, the smaller cluster sizes for the 40 practices and lower percentages receiving appropriate pharmacological therapy improved study efficiency, allowing us to address the research question and provide sufficient certainty to rule out the target 10% intervention effect. Further, among patients who did not receive appropriate pharmacological therapy, we were unable to determine if the GP did not consider that medications were indicated for other reasons or if the patient chose not to take the medications.

### Comparison with Previous Work

QI activities and CDS form an important part of Australian and international primary care policy, with the aim of optimizing the care provided to patients through the strengthening of clinical decision-making and improving care processes. There is increased focus on QI programs in Australian general practices, with payments to practices for participating in QI activities commencing in 2019 [31] and exploration of the use and regulation of CDS in general practice as the focus of current Australian government consultation [13].

The advent of EMRs has resulted in the development of QI and CDS software internationally and across care settings. Australian general practices were early adopters of EMRs in the 1990s, with near universal computerization by 2006 [21]. Similar to our trial, the results of trials of the use of QI dashboards and clinical decision support software (CDSS) tools in primary care to facilitate guideline-recommended prescribing have been underwhelming. For example, the TORPEDO cluster randomized trial in Australian primary care found that the proportion of patients with a recording of risk factors for CVD increased, but the proportion of patients at high CVD risk that received recommended prescriptions was not significantly different from the control group [29]. Hespe’s mixed methods descriptive study of a QI program in the Central and Eastern Sydney Primary Health Network area aimed to improve monitoring, prescribing practices, and attainment of BP and lipid targets for CVD risk reduction. The intervention included plan-do-study act cycles supported by a QI officer, workshops, an audit tool, and the HealthTracker guideline recommendation tool, but did not result in a change in the proportion of patients prescribed blood pressure and lipid-lowering medications over the 22-month intervention period.[24] The EBMeDS CDSS trial did not result in improvements in the management of diabetes in Belgian primary care despite high physician satisfaction [32].

Overall, the effectiveness of CDSS is generally small to moderate and highly variable [33] with the predictors of meaningful improvement unclear [34]. A systematic review of 11 trials incorporating clinical dashboards found limited evidence for their use, especially if they were not a component of a multifaceted intervention [35].

All of these studies highlight the challenges of implementation of QI dashboards and CDSS in general practice, including lack of time, practices’ need for technical support, a perceived lack of value for QI work, difficulty disseminating knowledge across the practice team, tensions between the team and clinical staff, workforce, a need for strong IT infrastructure and technical support, organization of practices as providing reactive (as opposed to proactive, planned) care and suboptimal recording of patient data in the EMR [24,32,36].

Our experiences with this trial have resulted in more robust mechanisms to validate our algorithms that incorporate the use of comprehensive data from Patron. We think this, combined with greater utility through supporting a wider range of decision support capabilities, may increase utilization of FHT and offer a greater chance of demonstrating the effectiveness of the FHT CDSS going forward. We will explore factors relevant to the FHT trial in a process evaluation and implementation study to be published separately. Upcoming studies of different conditions, including the results of the cancer module active control in this trial, will provide further information regarding the implementation and effectiveness of FHT in Australian general practice.

### Conclusions

In our trial, the CKD FHT intervention found no differences in trial primary or secondary outcomes except for a small improvement in prescribing of statin medication when initiation was recommended which may reduce future cardiovascular events. These results may be reflective of several factors, including the impact of the COVID-19 pandemic on general practice, technical implementation challenges, and sampling from high-performing practices. Which may have attenuated the intervention effect.

## Appendix

Multimedia Appendix 1 Screenshots of the Future Health Today dashboard and clinical decision support tool.

Multimedia Appendix 2 Primary, secondary, and health economics outcomes at 12 months postrandomization for quality improvement chronic kidney disease program.

Multimedia Appendix 3 CONSORT-eHEALTH checklist (V 1.6.1).

## Abbreviations

ACEI: angiotensin-converting enzyme inhibitor
ARB: angiotensin receptor blocker
CDS: clinical decision support
CDSS: clinical decision support software
CKD: chronic kidney disease
CPD: continuing professional development
CVD: cardiovascular disease
ECHO: Extension for Community Healthcare Outcomes
EMR: electronic medical record
FHT: Future Health Today
GEE: generalized estimating equation
GP: general practitioner
ICC: intracluster correlation
IRSD: Index of Relative Socioeconomic Disadvantage
MBS: Medicare Benefits Schedule
QI: quality improvement
SBP: systolic blood pressure

## Data Availability

The datasets generated and analyzed during this study are not publicly available due to them containing sensitive health information but may be available from the Department of General Practice and Primary Care, University of Melbourne subject to ethical and data governance approvals.
