# Supplementary material for: Effectiveness of Electronic Quality Improvement Activities to Reduce Cardiovascular Disease Risk in People With Chronic Kidney Disease in General Practice: Cluster Randomized Trial With Active Control
Source: JMIR Form Res. 2025 Feb 3;9:e54147. doi: 10.2196/54147 (PMC11833263; doi:10.2196/54147)
Supplement: Multimedia Appendix 1 [file formative_v9i1e54147_app1.pptx]

## Slide 1
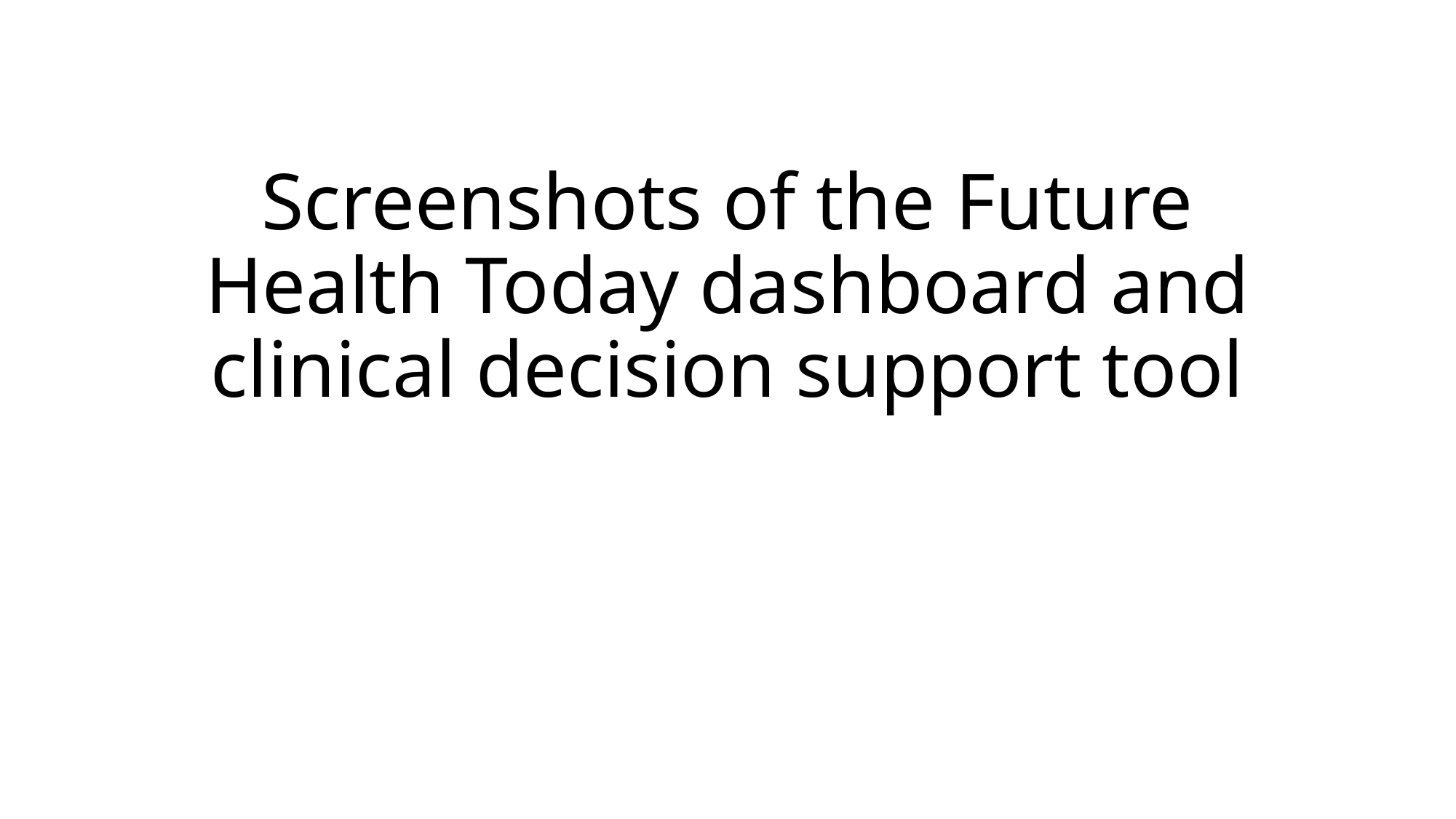

# Screenshots of the Future Health Today dashboard and clinical decision support tool

## Slide 2
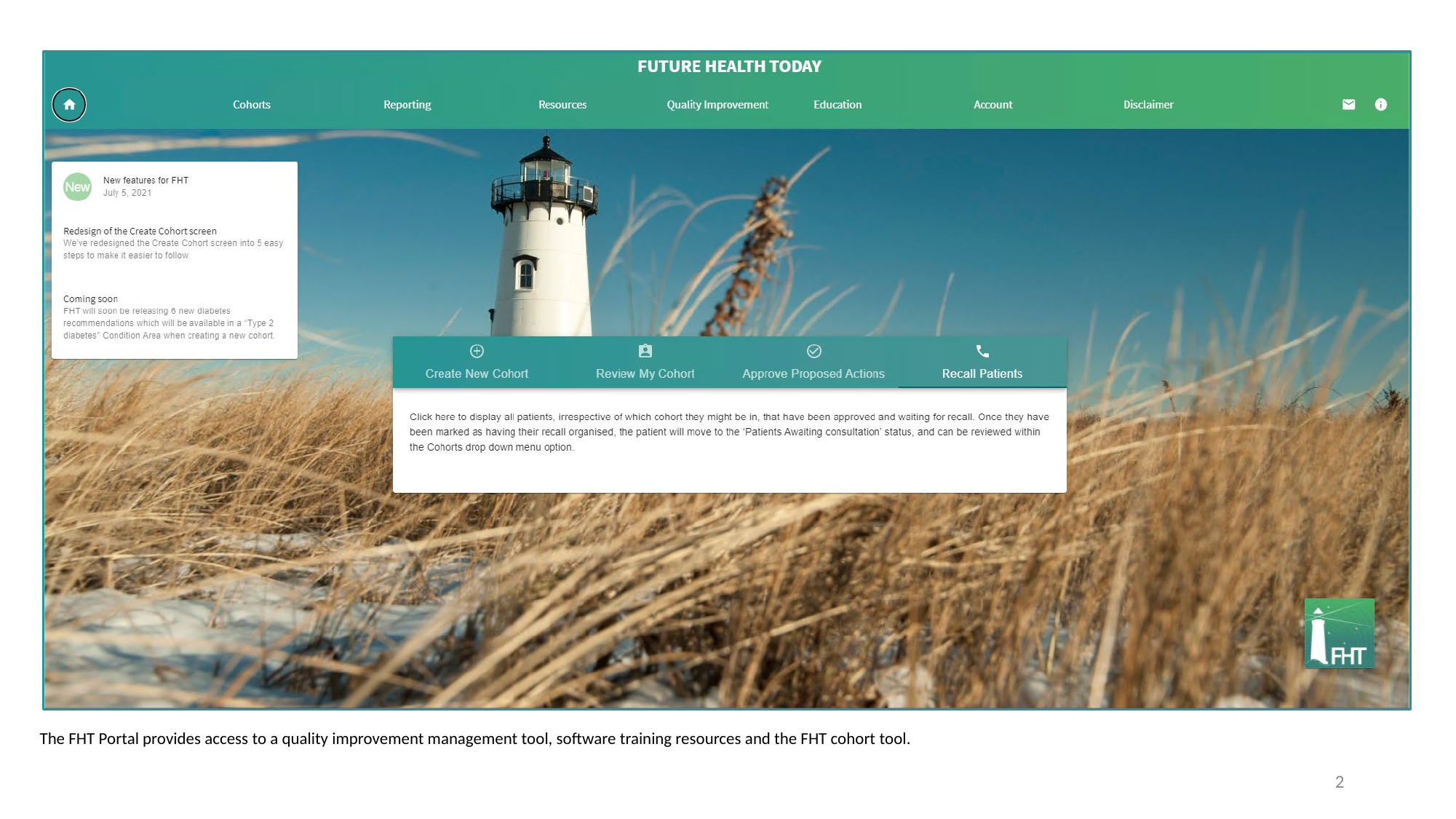

The FHT Portal provides access to a quality improvement management tool, software training resources and the FHT cohort tool.
2

## Slide 3
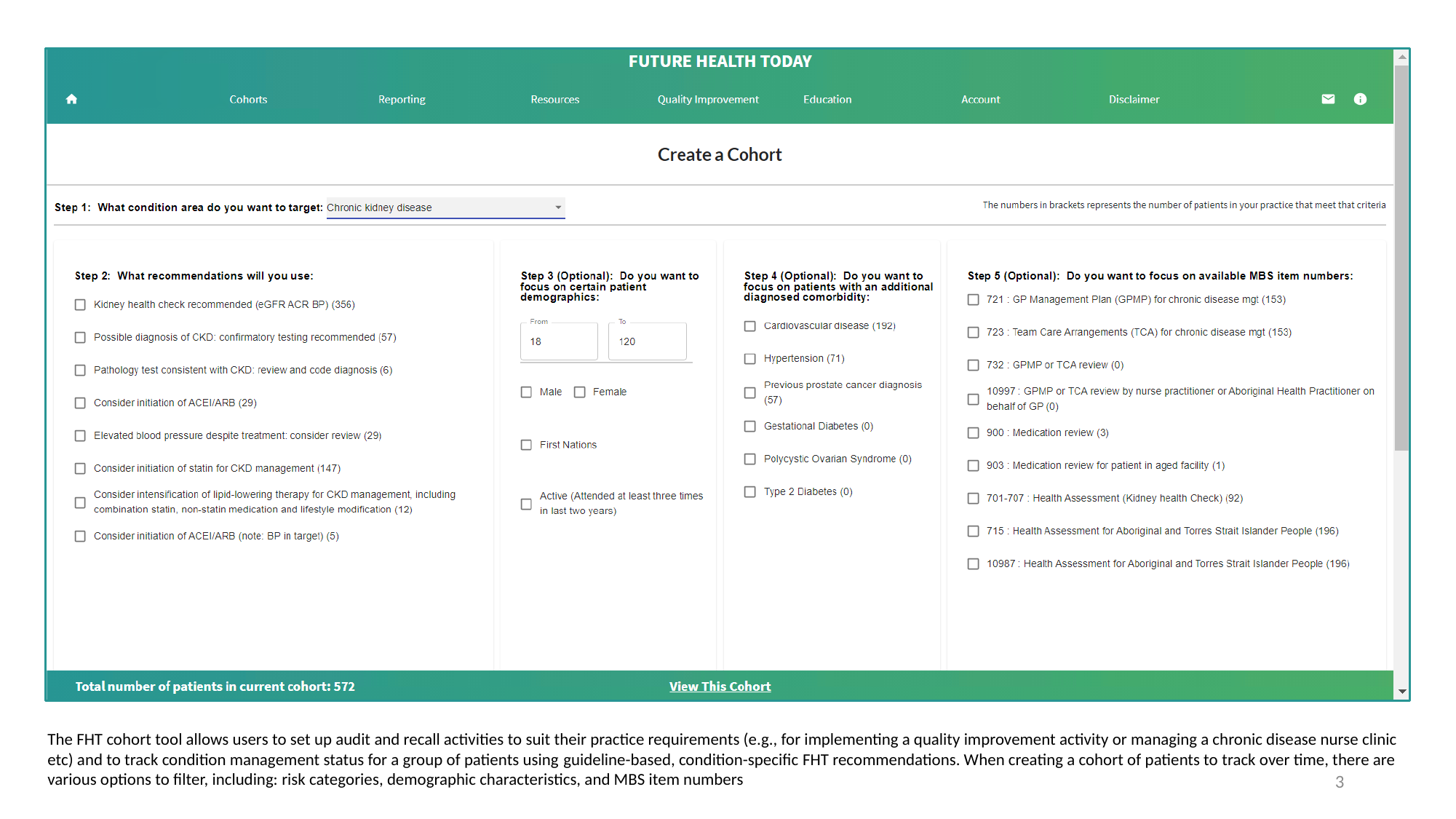

The FHT cohort tool allows users to set up audit and recall activities to suit their practice requirements (e.g., for implementing a quality improvement activity or managing a chronic disease nurse clinic etc) and to track condition management status for a group of patients using guideline-based, condition-specific FHT recommendations. When creating a cohort of patients to track over time, there are various options to filter, including: risk categories, demographic characteristics, and MBS item numbers
3

## Slide 4
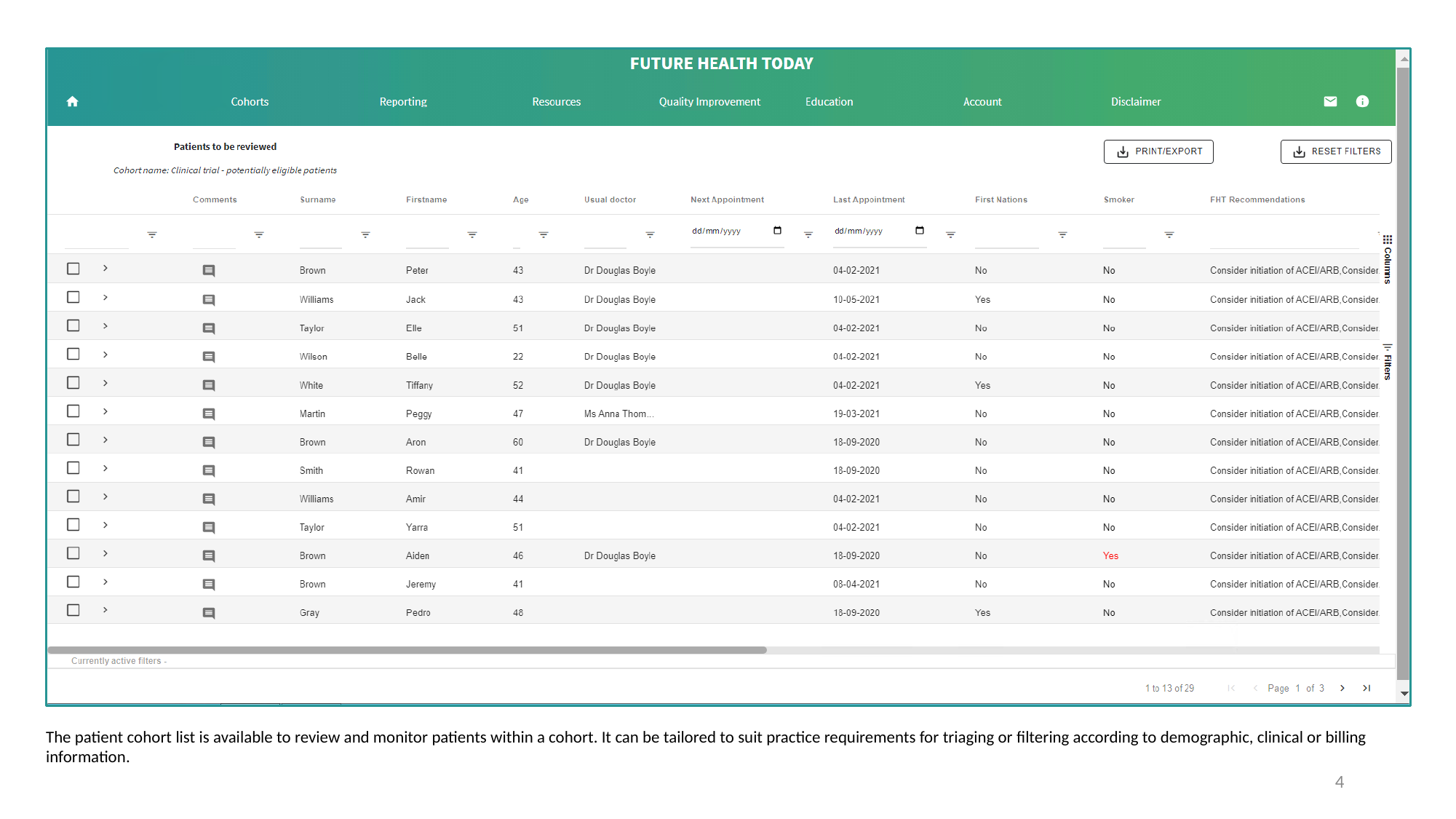

The patient cohort list is available to review and monitor patients within a cohort. It can be tailored to suit practice requirements for triaging or filtering according to demographic, clinical or billing information.
4

## Slide 5
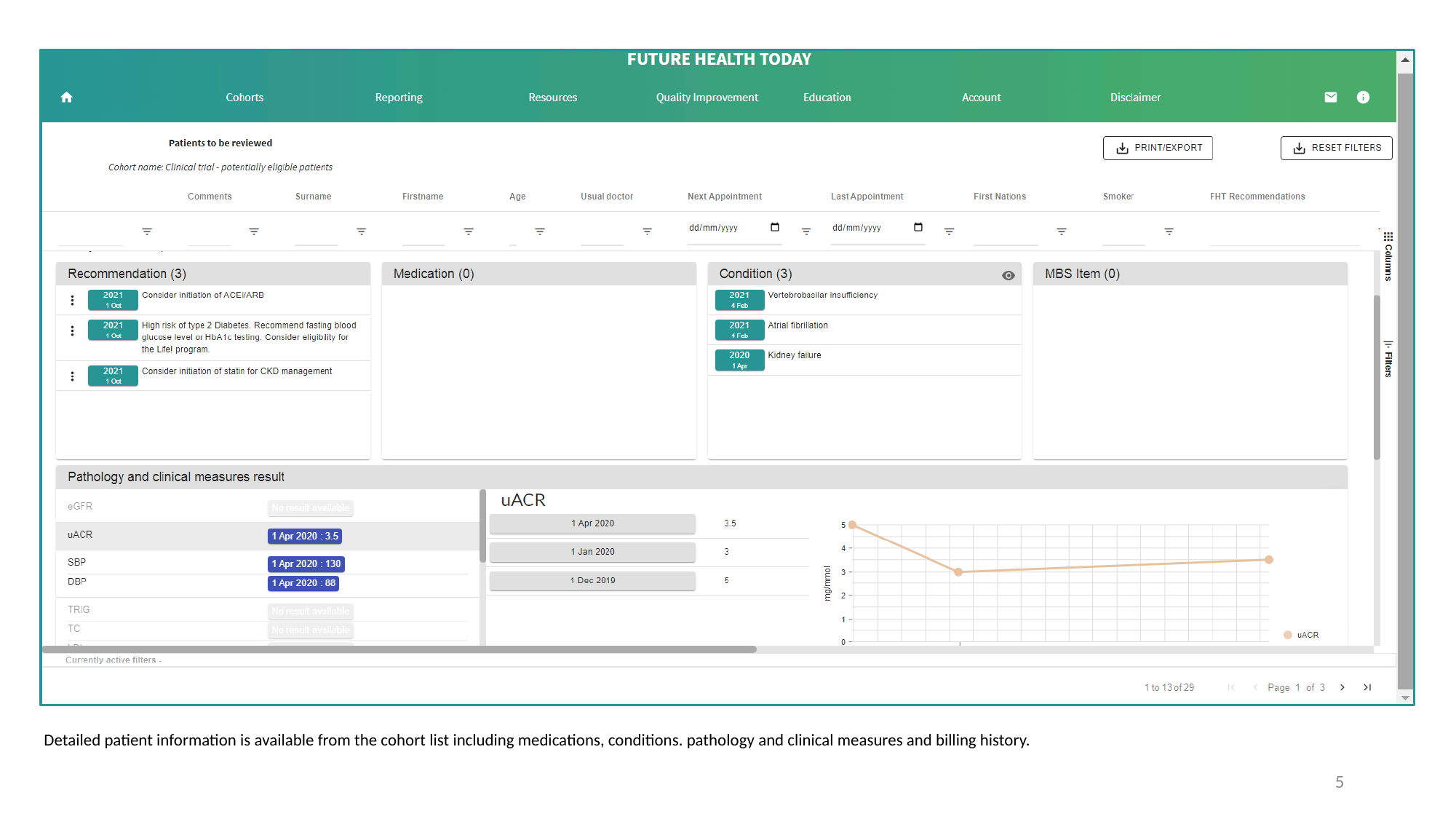

Detailed patient information is available from the cohort list including medications, conditions. pathology and clinical measures and billing history.
5

## Slide 6
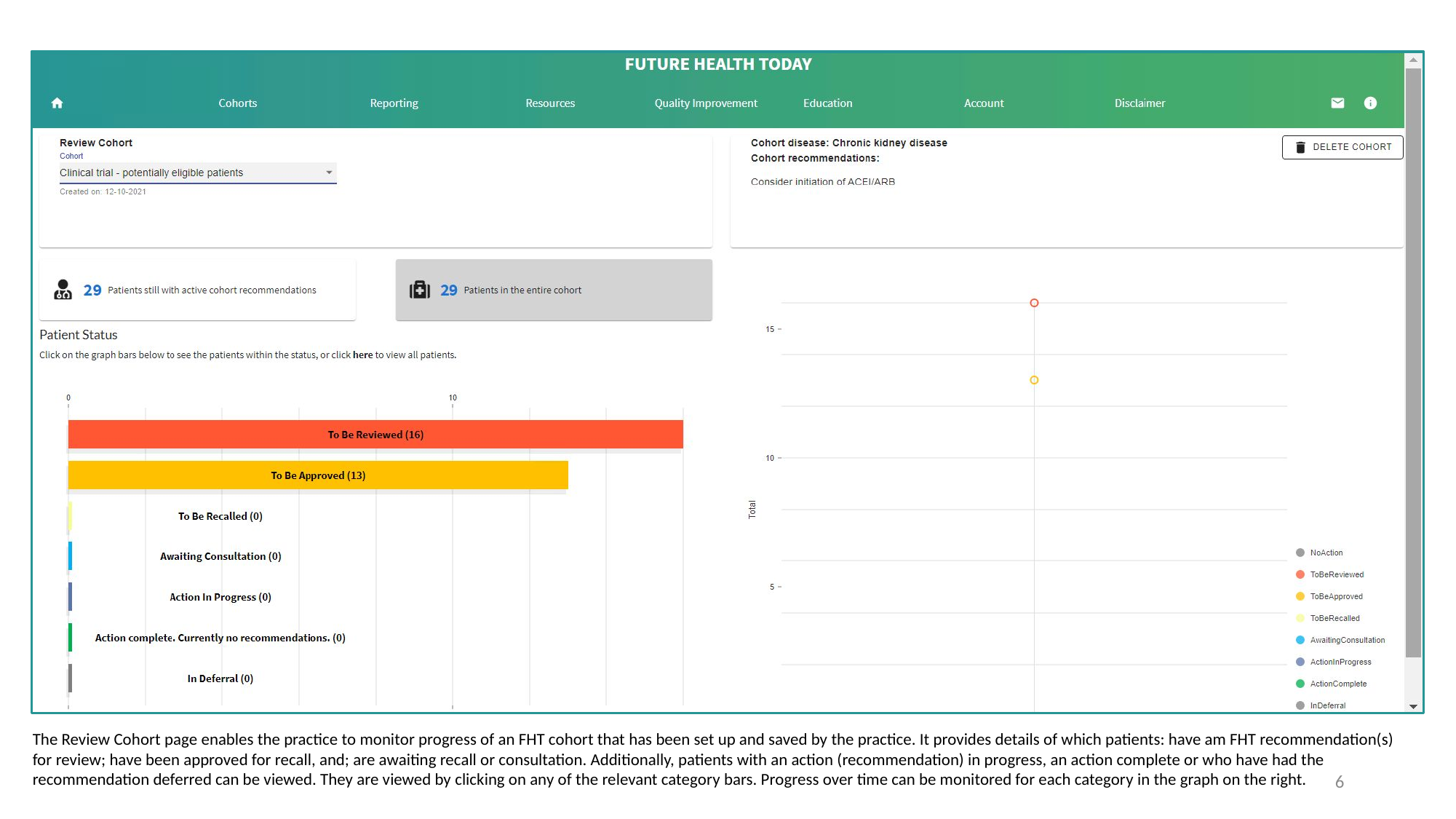

The Review Cohort page enables the practice to monitor progress of an FHT cohort that has been set up and saved by the practice. It provides details of which patients: have am FHT recommendation(s) for review; have been approved for recall, and; are awaiting recall or consultation. Additionally, patients with an action (recommendation) in progress, an action complete or who have had the recommendation deferred can be viewed. They are viewed by clicking on any of the relevant category bars. Progress over time can be monitored for each category in the graph on the right.
6

## Slide 7
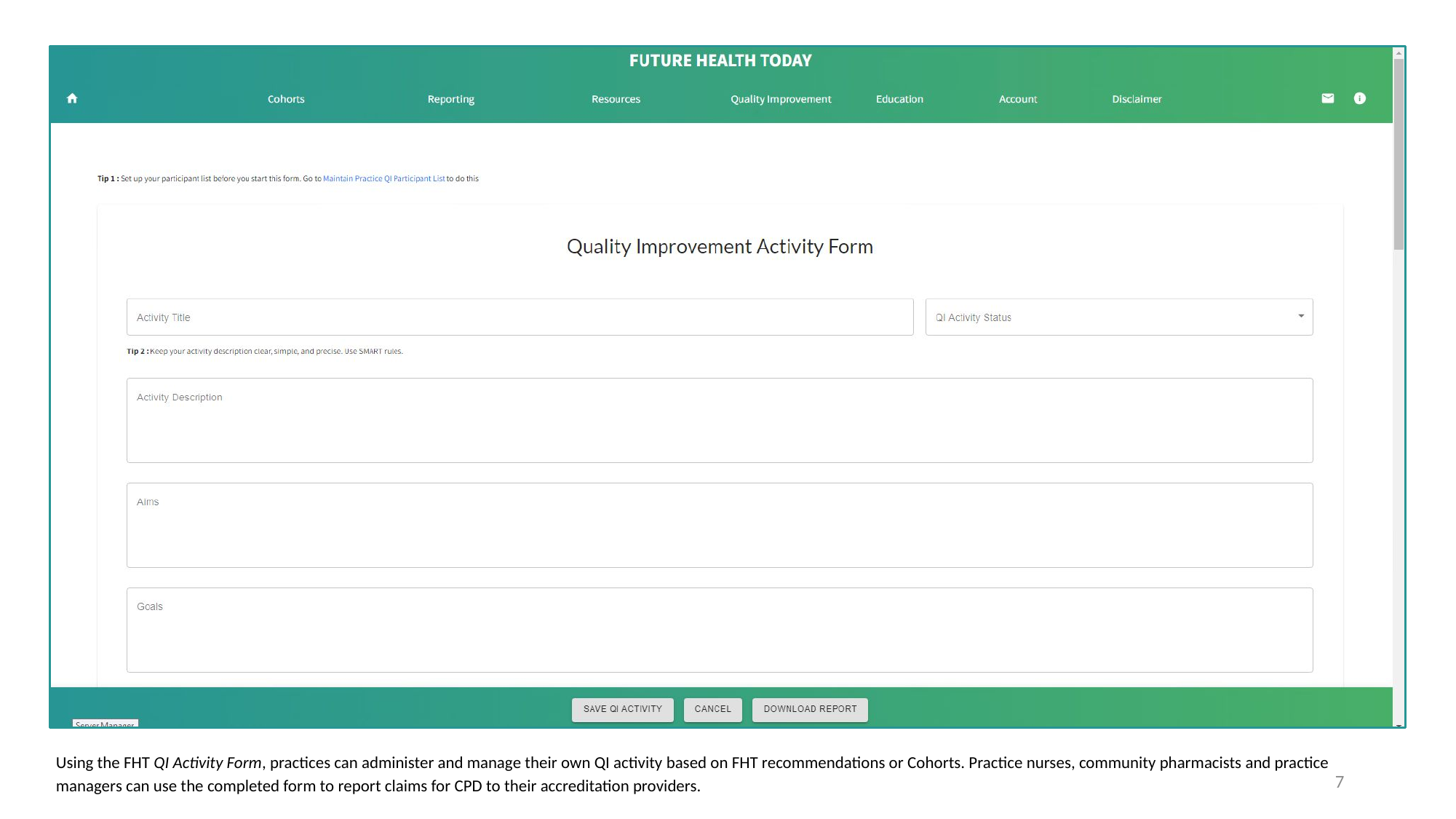

Using the FHT QI Activity Form, practices can administer and manage their own QI activity based on FHT recommendations or Cohorts. Practice nurses, community pharmacists and practice managers can use the completed form to report claims for CPD to their accreditation providers.
7

## Slide 8
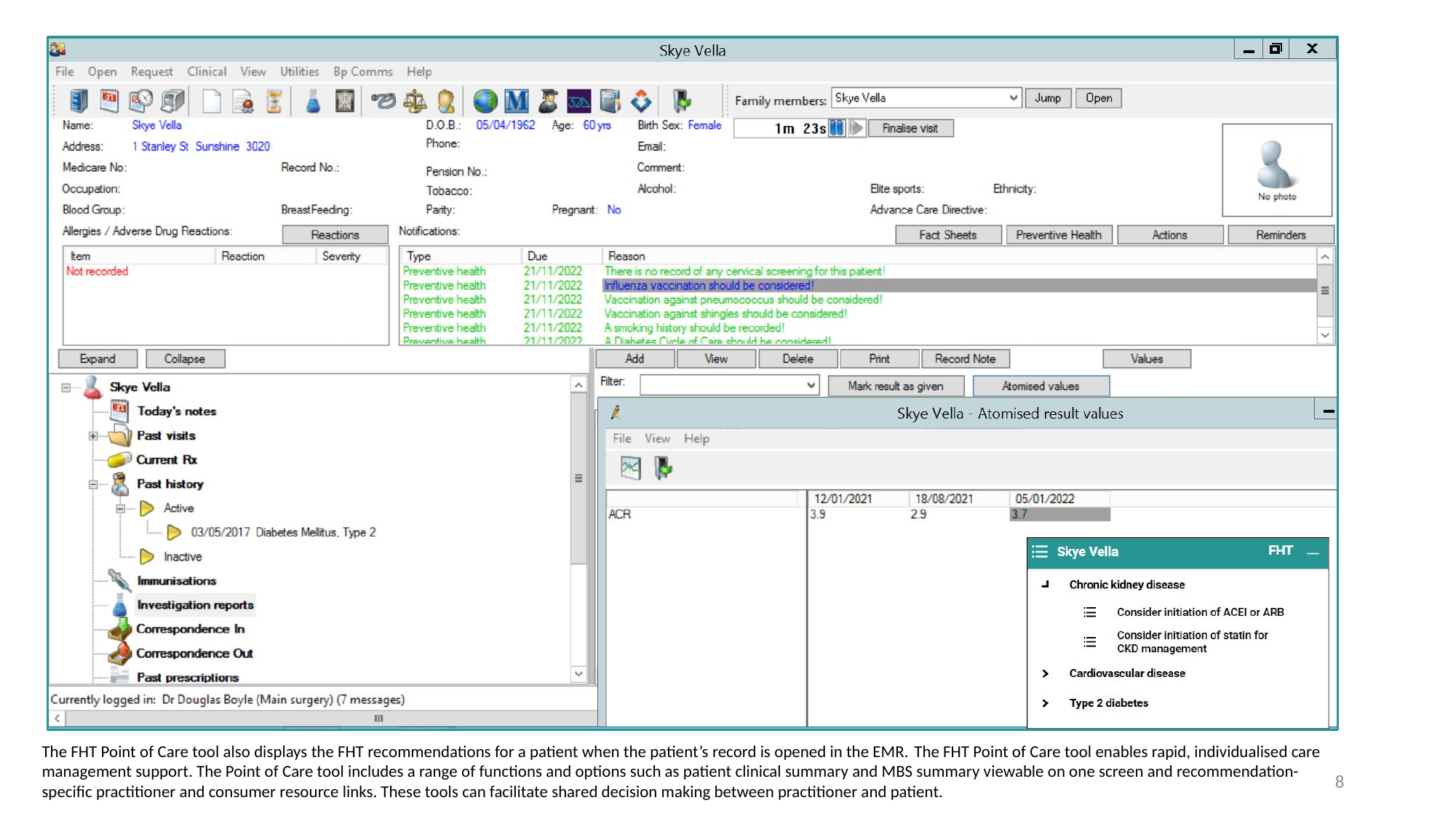

The FHT Point of Care tool also displays the FHT recommendations for a patient when the patient’s record is opened in the EMR. The FHT Point of Care tool enables rapid, individualised care management support. The Point of Care tool includes a range of functions and options such as patient clinical summary and MBS summary viewable on one screen and recommendation-specific practitioner and consumer resource links. These tools can facilitate shared decision making between practitioner and patient.
8
