# Supplementary material for: Effectiveness of Electronic Quality Improvement Activities to Reduce Cardiovascular Disease Risk in People With Chronic Kidney Disease in General Practice: Cluster Randomized Trial With Active Control
Source: JMIR Form Res. 2025 Feb 3;9:e54147. doi: 10.2196/54147 (PMC11833263; doi:10.2196/54147)
Supplement: Multimedia Appendix 2 [file formative_v9i1e54147_app2.docx]

Multimedia appendix 2: Primary, Secondary and Health Economics outcomes at 12 months post-randomisation for QI CKD Program

| **Estimand**  **Difference between QI CKD arm and the active control arm^1^ in the:** | **Method of estimation** | **Estimates**^4^ |
| --- | --- | --- |
| **Primary outcome** | | |
| Proportion of eligible patients with a diagnosis or pathology results consistent with CKD at baseline prescribed angiotensin converting enzyme (ACE) inhibitors or angiotensin receptor (AR) blockers and/or statins consistent with the RACGP Red Book [24], Kidney Health Australia (KHA) [5] and National Vascular Disease Prevention Alliance (NVDPA) [25 guidelines within the 12-month trial period. | Generalised linear model with the logit link (odds ratio) and identity link (difference in proportions) functions, respectively, and binomial distribution for both models. Both models included randomisation stratification factors, GP FTE and IRSD terciles as covariates, and used Generalised Estimating Equations with an exchangeable correlation structure and robust standard errors to allow for correlation of outcomes within general practice. | **Absolute measure**: Difference in proportions between the QI CKD arm and the active control arm  **Relative measure**: Odds ratio comparing the QI CKD arm and the active control arm. |
| **Secondary outcomes** | | |
| Proportion of patients with a diagnosis or pathology results consistent with CKD that are prescribed an ACE inhibitor or AR blocker consistent with the RACGP Red Book [24], KHA [5] and NVDPA guidelines [25] during the 12-month trial period. | Generalised linear model with the logit link (odds ratio) and identity link (difference in proportions) functions, respectively, and binomial distribution for both models. Both models included randomisation stratification factors, GP FTE and IRSD terciles, and recommendation to initiate statins as covariates, and used Generalised Estimating Equations with an exchangeable correlation structure and robust standard errors to allow for correlation of outcomes within general practice. | **Absolute measure**: Difference in proportions between the QI CKD arm and the active control arm  **Relative measure**: Odds ratio comparing the QI CKD arm and the active control arm |
| Proportion of patients with a diagnosis or pathology results consistent with CKD that are prescribed statin medication consistent with NVDPA guidelines [25] and ACC/AHA Guideline on the Primary Prevention of Cardiovascular Disease [36] during the 12-month trial period. | Generalised linear model with the logit link (odds ratio) and identity link (difference in proportions) functions, respectively, and binomial distribution for both models. Both models included randomisation stratification factors, GP FTE and IRSD terciles and recommendation to initiate ACEI/ARBs as covariates and used Generalised Estimating Equations with an exchangeable correlation structure and robust standard errors to allow for correlation of outcomes within general practice. | **Absolute measure**: Difference in proportions between the QI CKD arm and the active control arm  **Relative measure**: Odds ratio comparing the QI CKD arm and the active control arm |
| Mean change in systolic blood pressure (mmHg) between baseline^2^ and 12 months^3^ | Linear mixed effects model with study arm, randomisation stratification factors (GP FTE and IRSD terciles) and time (baseline and 12 months) as fixed effects, with a two-way interaction between arm and time, except for baseline where study arm means were constrained to be equal. Individuals and general practice were included as random effects. | **Absolute measure**: Difference in the mean change of the QI CKD arm and active control arm |
| Mean change in lipid results (mmol/L) between baseline^2^ and 12 months^3.^  Four types of lipid results were included:  Total cholesterol  LDL cholesterol  HDL cholesterol  Triglycerides | Linear mixed effects model with study arm, randomisation stratification factors (GP FTE and IRSD terciles) and time (baseline and 12 months) as fixed effects, with a two-way interaction between arm and time, except for baseline where study arm means were constrained to be equal. Individuals and general practice were included as random effects. | **Absolute measure**: Difference in the mean change of the QI CKD arm and active control arm |
| Mean change in urine albumin:creatinine ratio (uACR) between baseline^2^ and 12 months^3.^ | Linear mixed effects model with study arm, randomisation stratification factors (GP FTE and IRSD terciles) and time (baseline and 12 months) as fixed effects, with a two-way interaction between arm and time, except for baseline where study arm means were constrained to be equal. Individuals and general practice were included as random effects. | **Absolute measure**: Difference in the mean change of the QI CKD arm and active control arm |
| Proportion of patients with a ≥30% reduction in urine albumin:creatinine ratio between baseline^2^ and 12 months^3.^ | Generalised linear model with the logit link (odds ratio) and identity link (difference in proportions) functions, respectively, and binomial distribution for both models. Both models included randomisation stratification factors, GP FTE and IRSD terciles as covariates, and used Generalised Estimating Equations with an exchangeable correlation structure and robust standard errors to allow for correlation of outcomes within general practice. | **Absolute measure**: Difference in proportions the QI CKD arm and the active control arm  **Relative measure**: Odds ratio comparing the QI CKD arm and the active control arm |
| Proportion of people at low (<10%); moderate (10 to 15%) and high CVD risk (>15%) as per NVDPA [25] guidelines at 12-months. | Proportional odds logistic regression model with randomisation stratification factors (GP FTE and IRSD terciles) and baseline CVD risk group included as fixed effects. Generalised Estimating Equations with an exchangeable correlation structure and robust standard errors were used to allow for correlation of outcomes within general practice. | **Relative measure**: Cumulative odds ratio comparing the QI CKD arm and the active control arm |
| Mean change in glomerular filtration rate (ml/min/1.73m2) between baseline^2^ and 12-months^3^. | Linear mixed effects model with study arm, randomisation stratification factors (GP FTE and IRSD terciles) and time (baseline and 12 months) as fixed effects, with a two-way interaction between arm and time, except for baseline where study arm means were constrained to be equal. Individuals and general practice were included as random effects. | **Absolute measure**: Difference in the mean change of the QI CKD arm and active control arm |
| Rate of encounters per patient with a diagnosis or pathology results consistent with CKD at baseline in the 12-month trial period | Negative binomial mixed effects model with fixed effects for study arm and the randomisation stratification factors (GP FTE and IRSD terciles), and random effect for general practice. | **Absolute measure**: Difference in rates between the QI CKD arm and the active control arm  **Relative measure**: Rate ratio comparing the QI CKD arm and the active control arm |
| **Health Economics outcomes** |  |  |
| Proportion of patients receiving MBS benefits during the 12-month trial period | Logistic regression model adjusted for stratification factors (GP FTE and IRSD tercile), and robust standard errors to allow for correlation of outcomes within general practice. | **Absolute measure:** Difference in the proportion between the QI CKD arm and the active control arm  **Relative measure**: Odds ratio comparing the QI CKD arm and the active control arm |
| Cost to the government (AUD$, Medicare benefit payable) of primary care service utilisation per patient, per year during the 12-month trial period (conditional on receiving MBS benefits) | Generalised linear model with a log link and gamma distribution, adjusted for stratification factors (GP FTE and IRSD tercile) and clustering effect of general practice. | **Absolute measure:** Difference in mean dollars spent per patient per year between the QI CKD arm and the active control arm |

^1^ Target population were general practice patients with a diagnosis or pathology results consistent with CKD (see inclusion and exclusion criteria). Primary analysis was intention to treat (treatment policy for events occurring after randomisation, such as death or pregnancy).

^2^ Baseline measurement = the last measurement recorded in the patient’s electronic health record between the 4^th^ April 2021 (6 months prior to starting the trial) and when the trial started (4^th^ October 2021).

^3^ 12-month measurement = the last measurement recorded in the patient’s electronic health record occurring between the 4^th^ October 2021 to the 30^th^ September 2022 (12 month trial period). If there were no measurements the outcome was coded as missing.

^4^ Estimates of the intervention effect were reported with 95% confidence intervals and p-values.
